# On the Origin of the Treponematoses: A Phylogenetic Approach

**DOI:** 10.1371/journal.pntd.0000148

**Published:** 2008-01-15

**Authors:** Kristin N. Harper, Paolo S. Ocampo, Bret M. Steiner, Robert W. George, Michael S. Silverman, Shelly Bolotin, Allan Pillay, Nigel J. Saunders, George J. Armelagos

**Affiliations:** 1 Department of Population Biology, Ecology, and Evolution, Emory University, Atlanta, Georgia, United States of America; 2 School of Medicine, Emory University, Atlanta, Georgia, United States of America; 3 Laboratory Reference and Research Branch, Division of Sexually Transmitted Diseases Prevention, NCHHSTP, U.S. Centers for Disease Control and Prevention, Atlanta, Georgia, United States of America; 4 Department of Medicine, Division of Infectious Diseases, University of Toronto, Ontario, Canada; 5 Lakeridge Health Centre, Ontario, Canada; 6 Department of Microbiology, Mount Sinai Hospital, Toronto, Canada; 7 Sir William Dunn School of Pathology, University of Oxford, Oxford, United Kingdom; 8 Department of Anthropology, Emory University, Atlanta, Georgia, United States of America; Fundação Oswaldo Cruz, Brazil

## Abstract

**Background:**

Since the first recorded epidemic of syphilis in 1495, controversy has surrounded the origins of the bacterium *Treponema pallidum* subsp. *pallidum* and its relationship to the pathogens responsible for the other treponemal diseases: yaws, endemic syphilis, and pinta. Some researchers have argued that the syphilis-causing bacterium, or its progenitor, was brought from the New World to Europe by Christopher Columbus and his men, while others maintain that the treponematoses, including syphilis, have a much longer history on the European continent.

**Methodology/Principal Findings:**

We applied phylogenetics to this problem, using data from 21 genetic regions examined in 26 geographically disparate strains of pathogenic *Treponema*. Of all the strains examined, the venereal syphilis-causing strains originated most recently and were more closely related to yaws-causing strains from South America than to other non-venereal strains. Old World yaws-causing strains occupied a basal position on the tree, indicating that they arose first in human history, and a simian strain of *T. pallidum* was found to be indistinguishable from them.

**Conclusions/Significance:**

Our results lend support to the Columbian theory of syphilis's origin while suggesting that the non-sexually transmitted subspecies arose earlier in the Old World. This study represents the first attempt to address the problem of the origin of syphilis using molecular genetics, as well as the first source of information regarding the genetic make-up of non-venereal strains from the Western hemisphere.

## Author Summary

For 500 years, controversy has raged around the origin of *T. pallidum* subsp. *pallidum*, the bacterium responsible for syphilis. Did Christopher Columbus and his men introduce this pathogen into Renaissance Europe, after contracting it during their voyage to the New World? Or does syphilis have a much older history in the Old World? This paper represents the first attempt to use a phylogenetic approach to solve this question. In addition, it clarifies the evolutionary relationships between the pathogen that causes syphilis and the other *T. pallidum* subspecies, which cause the neglected tropical diseases yaws and endemic syphilis. Using a collection of pathogenic *Treponema* strains that is unprecedented in size, we show that yaws appears to be an ancient infection in humans while venereal syphilis arose relatively recently in human history. In addition, the closest relatives of syphilis-causing strains identified in this study were found in South America, providing support for the Columbian theory of syphilis's origin.

## Introduction

As Naples fell before the invading army of Charles the VIII in 1495, a plague broke out among the French leader's troops [1]. When the army disbanded shortly after the campaign, the troops, composed largely of mercenaries, returned to their homes and disseminated the disease across Europe [2],[3]. Today, it is generally agreed that this outbreak was the first recorded epidemic of syphilis. Although its death toll remains controversial, there is no question that the infection devastated the continent [4]. Because the epidemic followed quickly upon the return of Columbus and his men from the New World, some speculated that the disease originated in the Americas [2]. Indeed, reports surfaced that indigenous peoples of the New World suffered from a similar malady of great antiquity [5] and that symptoms of this disease had been observed in members of Columbus's crew [3]. In the twentieth century, criticisms of the Columbian hypothesis arose, with some hypothesizing that Europeans had simply not distinguished between syphilis and other diseases such as leprosy prior to 1495 [6].

It was soon recognized that different varieties of treponemal disease exist. Unlike syphilis, which is caused by the spirochete *T. pallidum* subspecies *pallidum*, the other types normally strike during childhood and are transmitted through skin-to-skin or oral contact. All are quite similar with regard to symptoms and progression [7], but endemic syphilis, or bejel, caused by subsp. *endemicum*, has historically affected people living in hot, arid climates and yaws, caused by subsp. *pertenue*, is limited to hot and humid areas. Pinta, caused by *Treponema carateum*, is the most distinct member of this family of diseases. Once found in Central and South America, this mild disease is characterized solely by alterations in skin color. Today, the debate over the origin of treponemal disease encompasses arguments about whether the four infections are caused by distinct but related pathogens [8] or one protean bacterium with many manifestations [9].

Paleopathologists have played a pivotal role in addressing the question surrounding the origin of syphilis. The treponemal diseases, with the exception of pinta, leave distinct marks upon the skeleton and can thus be studied in past civilizations. Paleopathological studies of populations in the pre-Columbian New World show that treponemal disease was prevalent, with cases dating back 7,000 years and increasing over time [10]. In contrast, paleopathological studies of large pre-Columbian populations in Europe and Africa have yielded no evidence of treponemal disease [11]–[13]. However, isolated cases of pre-Columbian treponemal disease from other Old World excavation sites have been reported sporadically [14]. Although these cases have often been met with criticism regarding diagnosis, dating, and epidemiological context, they have convinced some that treponemal disease did exist in the pre-Columbian Old World [15],[16].

The *T. pallidum* genome is small (roughly 1,000 kilobases) and was sequenced in 1998 [17]. However, comparative genetic studies of *T. pallidum* [18]–[23] have been rare and relatively small in scope. One reason for this is the difficulty in obtaining non-venereal strains for study. Today only five known laboratory strains of subsp. *pertenue*, two strains of subsp. *endemicum*, and no strains or samples of *T. carateum* survive. Furthermore, it is uncertain whether the disease pinta still exists. No cases have been reported to the World Health Organization from the former endemic countries Mexico or Colombia since 1979 [24]. Similarly, endemic syphilis was eradicated some time ago in its European focus, Bosnia [25]. In Turkey, only one infected family has been reported in the last forty years [26], and a large survey in the United Arab Emirates revealed only non-active cases of endemic syphilis in the elderly [27]. In the Old World, yaws is still reported but appears limited to a few isolated foci in the Republic of Congo and the Democratic Republic of Congo [28],[29], as well as Indonesia and Timor-Leste, where roughly 5,000 cases are reported annually [30]. In the New World, yaws appears to be constrained to an ever-constricting area of Guyana's interior [31]. Because of the paucity of samples available for experiments, most comparative studies have included very few non-venereal strains.

Another limitation on comparative studies has been the small amount of variation present in the *T. pallidum* genome. Variability is sufficiently low that the discovery of a single nucleotide polymorphism (SNP) has warranted publication in the past [18],[20],[21]. One study has suggested that most variation between the subsp. *pallidum* and *pertenue* genomes lies within the *tpr* gene family [32], a family of 12 genes with sequence similarity that make up roughly 2% of the genome. A recent examination of this large gene family demonstrated more extensive variation between strains than had previous studies but also documented an unusually high frequency of intra-gene conversion events [23]. Thus, it is possible that most polymorphism in the *T. pallidum* genome may be concentrated in genes with limited phylogenetic informativeness.

Our goal in this study was to identify variable sites in the *T. pallidum* genome and characterize them in as many non-venereal strains as possible, in order to test the hypothesis that syphilis emerged in humankind's recent past, from New World-derived strains of *T. pallidum*. Recombination can result in a phylogeny different from the true one [33],[34], and it has been shown that gene conversion is an important evolutionary mechanism in one large *T. pallidum* gene family [23]. For this reason, after sequencing many sites from around the genome, we performed rigorous tests for recombination, then built a phylogeny from the non-recombining SNPs and insertions/deletions (indels) identified. These results, paired with geographic analysis of strains, provide novel information on the history of *T. pallidum.*

## Methods

### Origin and Preparation of Isolates

Twenty-two human *Treponema pallidum* strains, one *T. pallidum* strain collected from a wild baboon, and three *T. paraluiscuniculi* strains, which are responsible for venereal syphilis in rabbits, were used in this study (Table 1). This included all laboratory strains of subsp. *pertenue* (*n* = 5) and subsp. *endemicum* (*n* = 2). Guyana is the only known active site of yaws infection in the western hemisphere. In order to provide representation of non-venereal strains from the Americas, subsp. *pertenue* strains (*n* = 2) were obtained from indigenous children with clinical evidence of non-venereal treponemal disease during a humanitarian medical mission to protected native reserves deep within the Guyanese interior. These samples were collected from a population that has had very little contact with the outside world, both due to the remoteness of their forest location and to legal restrictions on outsider interference. Ethical approval for the sample collection protocol was obtained from the Ethics Board at Lakeridge Health Centre (Oshawa, Canada) and included obtaining informed consent from patients. Scrapings were taken from active yaws lesions and were immediately deposited in either ethanol or saline. They were kept as cool as possible, but not frozen, for the duration of the two-week medical trip. Upon return to the laboratory, they were kept frozen until DNA was extracted. Additional subsp. *pertenue* samples (*n* = 4) came from a Dutch collection of strains destroyed years ago during a freezer breakdown. Although these strains were non-viable, the organisms were kept and a small amount of each was made available for this project. Eleven syphilis strains were chosen for analysis based on geographic and chronological span. This included two strains of uncertain subspecies and origin, Haiti B and Madras, which were originally labeled as subsp. *pertenue* strains, but appear to be subsp. *pallidum* strains based on both genetic studies and clinical manifestations in a rabbit model [18], [20]–[22],[35].

**Table 1:** *Treponema sp.* analyzed in this study (N = 26).

Strain Name	Subspecies	Place Collected	Date Collected	Sample Type	Source
Brazzaville	*pertenue*	Congo	1960	DNA only	L. Schouls
CDC-1	*pertenue*	Nigeria	1980	isolate	CDC
CDC-2	*pertenue*	Nigeria	1980	isolate	CDC
CDC-2575	*pertenue*	Ghana	1980	DNA only	L. Schouls
Gauthier	*pertenue*	Nigeria	1960	isolate	S. Lukehart
Ghana	*pertenue*	Ghana	1988	DNA only	L. Schouls
Guyana 1	*pertenue*	Guyana	2005	DNA only	This Study
Guyana 2	*pertenue*	Guyana	2005	DNA only	This Study
Pariaman	*pertenue*	Indonesia	1988	DNA only	L. Schouls
Samoa D	*pertenue*	Samoa	1953	isolate	CDC
Samoa F	*pertenue*	Samoa	1953	isolate	CDC
Bosnia A	*endemicum*	Bosnia	1950	isolate	CDC
Iraq B	*endemicum*	Iraq	1951	isolate	CDC
Chicago B	*pallidum*	Chicago	1951	isolate	CDC
Dallas 1	*pallidum*	Dallas	1991	isolate	CDC
Grady	*pallidum*	Atlanta	1998	isolate	CDC
Haiti B*	*pallidum*	Haiti	1951	isolate	S. Lukehart
					R. Limberger
Madras*	*pallidum*	India	1954	isolate	CDC
Mexico A	*pallidum*	Mexico	1953	isolate	CDC
Nichols	*pallidum*	Wash, D.C.	1912	isolate	CDC
Philadelphia 1	*pallidum*	Philadelphia	1988	isolate	CDC
South Africa	*pallidum*	South Africa	1998	DNA only	A. Pillay
Simian Strain	?	Guinea	1962	isolate	S. Lukehart
*T. paraluiscuniculi Strain A*	N/A	?	?	isolate	CDC
*T. paraluiscuniculi Strain H*	N/A	?	?	isolate	S. Lukehart
*T. paraluiscuniculi Strain M*	N/A	?	?	isolate	S. Lukehart

*: Strains were originally labeled subsp. *pertenue*, but various studies suggest they are, in reality, subsp. *pallidum* strains.

DNA was obtained from *Treponema* organisms grown in rabbit tissue or from clinical specimens. Laboratory isolates of *T. pallidum* were grown in New Zealand white rabbits, consistent with guidelines set out by the Institutional Animal Care and Use Committee at the U.S. Center for Disease Control and Prevention (CDC). DNA was isolated using the QIAamp mini kit (Qiagen, Valencia, CA) according to the manufacturer's instructions for tissue or fluid preparations, depending on the type of sample.

Whole genome amplification (REPLI-g Midi Kit, Qiagen) was performed on the strains for which only a limited amount of DNA was available: the four subsp. *pertenue* strains from the Dutch collection and a *pallidum* strain obtained from a South African clinical specimen. The whole genome amplification product was used as a template for subsequent polymerase chain reactions (PCRs). Unfortunately, amplification of the whole genome of the two strains collected in Guyana could not be performed, due to DNA degradation.

### Sequencing

**Figure 1:**
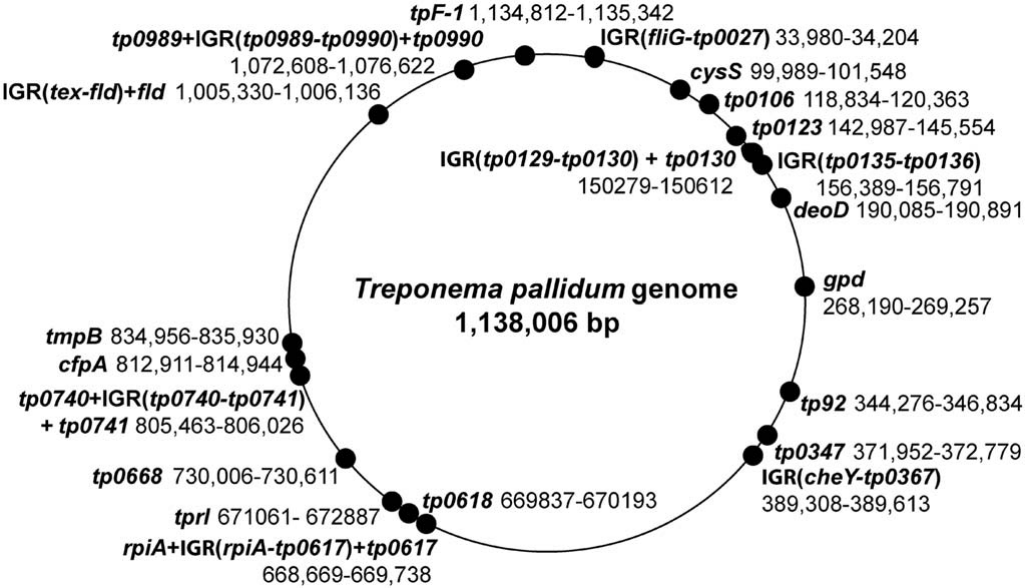
Genome coordinates of the 21 regions targeted for sequencing. The name of the genes and/or intergenic regions (IGRs) amplified are shown in bold next to their numeric coordinates and a point denoting their location in the genome. The coordinates of the nucleotides sequenced within each gene/IGR can be found in Table 2.

Twenty-one genetic regions (Table 2) were sequenced in all strains except for the two clinical samples from Guyana. These regions were scattered around the genome (Fig. 1), and were chosen based on previously demonstrated polymorphism [18], [20]–[22],[36], implication in pathogenesis, or because they harbored repetitive sequences. Because a very limited amount of considerably degraded DNA was available from the clinical specimens collected in Guyana, only seven polymorphic sites encompassing 17 SNPs could be sequenced in these strains. The sites were chosen based on 1) which appeared to be the most phylogenetically informative at the time of sequencing; and 2) which involved small molecular weight products, easily amplified from damaged DNA. They included IGR(*fliG-tp0027*), *deoD, gpd*, *tp0618*, *tprI*, *cfpA,* and *tpF-1*.

**Table 2:** The 21 genetic regions sequenced in this study.

Genetic Region^1^	Description/Function^2^	Amplicon Size **(Region Sequenced)** 3	Accession Numbers
**IGR(** ***fliG-tp0027*** **)**	Intergenic Region	**280 bp** (1–207)	EU101881–EU101906
***cysS (tp0091)***	Cysteinyl-tRNA synthetase	**168 bp** (440–607)	EU101781–EU101804
***tp0106***	Transporter (possible carnitine, choline, or glycine betaine transporter)	**598 bp** (55–652)	EU102099–EU102122
***tp0123***	Hypothetical protein containing TPR domains	**596 bp** (253–809)	EU102123–EU102146
		**234 bp**	
**IGR(** ***tp0129-tp0130*** **)**	Intergenic Region	(158–334)	EU101955–EU101978
***tp0130***	Hypothetical protein, basic	(361–417)	
**IGR(** ***tp0135-tp0136*** **)**	Intergenic Region	**301 bp** (24–324)	EU101979–EU102002
***deoD (tp0170/pfs)***	Purine nucleoside phosphorylase	**501 bp** (482–804)	EU101805–EU101830
***gpd*** ** (** ***tp0257/glpQ*** **)**	Glycerophosphodiester phosphodiesterase	**331 bp**(366–633)	EU101831–EU101856
***tp92*** ** (** ***tp0326*** **)**	Predicted outer membrane protein	**1030 bp** (1530–2504)	EU102075–EU102098
***tp0347***	Hypothetical protein	**340 bp** (1–228)	EU102147–EU102170
**IGR(** ***cheY-tp0367*** **)**	Intergenic Region	**165 bp** (1–160)	EU101857–EU101880
		**333 bp**	
***rpiA (tp0616)***	Ribose 5-phosphate isomerase	(546–762)	EU101907–EU101930
***IGR(rpiA-tp0617)***	Intergenic Region	(1–32)	
***tp0617***	Hypothetical protein, basic	(193–276)	
***tp0618***	Hypothetical protein, acidic	**274 bp** (236–347)	EU102171–EU102194
***tprI*** ** (** ***tp0620*** **)**	Member of the *Treponema pallidum* repeat family	**376 bp** (1–162)	EU102245–EU102267
***tp0668***	Conserved hypothetical protein (integral membrane)	**243 bp** (256–498)	EU102195–EU102218
		**223 bp**	
***tp0740***	Conserved hypothetical protein	(1–42)	EU102003–EU102026
**IGR(** ***tp0740-tp0741*** **)**	Intergenic Region	(1–130)	
***tp0741***	Conserved hypothetical protein	(562–612)	
***cfpA*** ** (** ***tp0748*** **)**	Cytoplasmic filament protein	**556 bp**(23–533)	EU101755–EU101780
***tmpB (tp0769)***	Predicted outer membrane protein	**966 bp** (57–919)	EU102051-EU102074
		**551 bp**	
**IGR(** ***tex-fld*** **)**	Intergenic Region	(1–369)	EU101931–EU101954
***fld (tp0925)***	Flavodoxin	(1–101)	
		**150 bp**	
***tp0989***	P26 related protein	(1–39)	EU102027–EU102050
***IGR(tp0989-tp0990)***	Intergenic Region	(1–46)	
***tp0990***	Conserved hypothetical protein	(1–65)	
***tpf-1*** ** (** ***tp1038*** **)**	Antigen Tpf-1	**171 bp** (81–190)	EU102219–EU102244

1 Genes are identified by both commonly used names, when present, and the gene number in the *T. pallidum* genome. Intergenic regions (IGRs) are identified by the genes between which they fall. Where amplicons contained multiple genetic regions, they are listed on separate lines.

2 As listed on the STDgen website (http://stdgen.northwestern.edu/).

3 The size of the amplicon, in basepairs, is listed in bold. Below, the coordinates of the region sequenced within the gene is given in nucleotide positions.

Primers (Table S1) were designed using the programs MacVector (Accelrys, Burlington, MA) and Primer3 [37] and the subsp. *pallidum* genomic DNA sequences posted on the Los Alamos National Laboratory Bioscience Division's STD Sequence Databases webpage (http://www.stdgen.northwestern.edu). PCR amplifications were performed in 50 µL reactions containing 0.5 µM primers (Invitrogen, Carlsbad, CA), 200 µM GeneAmp dNTPs (Applied Biosystems, Foster City, CA), and 2.5 U AmpliTaq Gold polymerase with Gold Buffer and 3.0 mM MgCl_2_ (Applied Biosystems). PCR conditions were as follows: One cycle of 94°C for 5 minutes; 35 cycles of 94°C for 30 seconds, primer annealing at the appropriate temperature for 30 seconds (Table S1), and 72°C for 1 minute and 30 seconds; followed by a final extension for seven minutes at 72°C. Standard precautions to avoid DNA contamination were employed, including the use of negative controls, aerosol resistant pipette-tips, and a three-station PCR set-up protocol. In addition, sequences that were especially important in the phylogenetic analysis were confirmed through independent amplifications. Amplicons were purified using a gel extraction kit (Qiagen, Valencia, CA) and sequenced using either an ABI 3100/3700 Automated Capillary DNA Sequencer and the Big Dye Sequencing kit (Applied Biosystems) or the CEQ 8000 Genetic Analysis System and the DTCS Quick Start Kit (Beckman-Coulter, Fullerton, CA). Sequencing was performed at the CDC, SeqWright/Fisher Sequencing Services, or Oregon Health Sciences University's Core Laboratory. Sequences were deposited in GenBank under the accession numbers listed (Table 2).

### Sequence Analysis

Open reading frames containing polymorphisms were translated into protein sequences to examine the resulting amino acid changes. The average number of nucleotide differences between groups, as well as the average pairwise difference for each polymorphic gene, was calculated using DnaSP [38]. All nucleotide substitutions occurring in genic areas were deemed either synonymous or non-synonymous (Table S2). The amino acid substitutions resulting from non-synonymous changes were scored according to three criteria. Amino acids were categorized according to charge, polarity and volume, and Grantham's distance [39],[40]. In the first two cases, a substitution resulting in a change from one category to another was considered a radical substitution, while others were considered conservative. In the latter case, substitutions resulting in distances greater than 100, according to Grantham's index, were categorized as radical, others conservative. Substitutions considered radical in at least two of the three categories were scored as radical (Table S2). Secondary DNA structure in the polymorphic region of IGR(*fliG-tp0027*) was predicted using the program Mfold [41].

### Phylogenetic Analysis

In order to rule out recombination in the areas analyzed, each gene was tested against all of the paralogs present in the sequenced *T. pallidum* subsp. *pallidum* Nichols strain genome, using the program RDP2 [42]. This type of search would identify recombination events between the strains sequenced in this study and the paralogs present in the sequenced genome, as well as recombination between the genes in the strains sequenced. In addition, when small stretches of extremely polymorphic DNA were identified, a BLAST search was performed in order to identify possible donor regions involved in intragenomic conversion events (http://www.ncbi.nlm.nih.gov/BLAST/). Because the complete genome sequence was only available for one strain, it is possible that some recombination events, between paralogs or donor regions not present in the sequenced genome, would not be detected. For this reason, the frequency of synonymous and non-synonymous substitutions was examined in highly polymorphic stretches; regions with a high number of synonymous substitutions and multiple substitutions per codon were considered to be possible results of recombination and were excluded from the analysis. The sequence of these regions can be found in Table S3. The complete alignments, constructed as described below and encompassing all polymorphism in which within-gene recombination had been ruled out, were also analyzed using RDP2, in order to rule out large-scale genomic recombination.

In order to construct phylogenetic trees incorporating all variation, an alignment of the concatenated SNPs and indels was created using ClustalX version 1.83 [43]. The order of the regions in the concatenation corresponds to their position in the genome. Modeltest [44] was used to choose the appropriate model of nucleotide substitution, Kimura's two parameter, and phylogenetic trees were built in *PAUP 4.0 [45]. Both maximum likelihood and maximum parsimony methods were employed, to glean the most information from both parsimony-uninformative traits and indels, respectively. *T. paraluiscuniculi* was used as an outgroup (i.e. a taxon known to lie outside of the *T. pallidum* grouping) with which to root the trees and determine the directionality of substitutions. In the alignment, indels were trimmed to one basepair, in order to prevent their greater length from dominating the analysis. One thousand replicates were run to obtain bootstrap support at each node, with starting trees obtained through random, step-wise addition. Tree bisection and reconnection was used for the branch-swapping algorithm. The maximum likelihood tree was chosen for display, with bootstrap support from both methods displayed at nodes. Trees in which trimmed indels were weighted 1/5 the value of substitutions were also created, in light of the faster mutation rate of repeat regions. Because many substitutions were contained in one gene, *tp92*, trees in which polymorphism in *tp92* was not considered were built. In addition, trees built only from synonymous SNPS or non-synonymous SNPs were created using the methods described above.

## Results

### Sequence Variation

Of the twenty-one genes sequenced in this study, data from *T. paraluiscuniculi* was obtained for all but one gene, *tprI*, which had previously been shown to be absent from the genome of this species [46]. The *tp0618* gene could be amplified from *T. paraluiscuniculi* Strain A, but not from Strains H and M. It is possible that the gene is missing from the genome of the latter two strains or that the sequence in the priming regions has diverged sufficiently to prevent amplification.

No areas likely to result from recombination were identified in formal tests performed in RDP2. However, polymorphic regions in two genes, *tp0618* and *tp92*, contained small tracts of highly variable DNA with an elevated frequency of synonymous nucleotide substitutions and multiple substitutions per codon. No possible donor regions could be identified for these tracts using BLAST searches on the *T. pallidum* genome. However, because such a high frequency of synonymous substitutions could be explained by recombination, and it was thus not clear how many mutational events were responsible for the polymorphism observed, the polymorphism in *tp0618* and four polymorphic regions from *tp92* were excluded from the phylogenetic analysis. The *tp92* substitutions included in the analysis can be found in polymorphism Table S2, while all substitutions can be found in polymorphism Table S3.

In the remaining 7 kilobases of the *T. pallidum* genome examined, which were sequenced from 20 scattered regions, a total of 70 SNPs and 12 indels were identified. Most of them are described here for the first time. Twenty-six substitutions occurred between *T. pallidum* strains (Table 3), amounting to about one substitution per 275 basepairs. This value is likely to significantly overestimate the amount of polymorphism typical of the genome, however. A number of regions were sequenced either because of their previously demonstrated polymorphism or because they were thought likely to contain variation. This may have weighted the regions sampled towards exceptionally polymorphic areas. The region sequenced in *tp92*, for example, contained 7 of the 24 substitutions observed between *T. pallidum* strains (Table 3). Roughly two-thirds of the total substitutions observed represented fixed differences between *T. pallidum* and the outgroup, *T. paraluiscuniculi* (Table 4). Singletons were rare, accounting for only 7 of the 70 observed substitutions (Table S2). Three of these singletons were found in the Pariaman strain, which was geographically distinct from other subsp. *pertenue* strains, and no singletons were observed in the other whole genome amplified strains. Thus, it appears that whole genome amplification did not introduce spurious substitutions.

**Table 3:** Summary of polymorphism found between *T. pallidum* strains.

Genetic Region	Size of Sequenced Fragment (bp)	# Indels	# Polymorphic Sites	# Sites with NS Substitutions	# Parsimony Informative Sites^1^	π			π S: π NS
						All Sites	S Sites	NS Sites	
**IGR(** ***fliG-tp0027*** **)**	207	1	1	-	2	-	-	-	-
***tp0106***	598	0	1	0	0	0.00016	0.00061	0.00000	-
***tp0123***	557	0	1	1	0	0.00017	0.00000	0.00023	0
**IGR(** ***tp0135-tp0136*** **)**	301	1	0	-	1	-	-	-	-
***deoD***	323	0	1	0	1	0.00160	0.00611	0.00000	-
***gpd***	268	0	1	0	1	0.00193	0.00823	0.00000	-
***tp92*** 2	975	1	7	7	6	0.00194	0.00000	0.00257	0
***tp0347***	228	1	1	0	2	0.00082	0.00329	0.00000	-
**IGR(** ***cheY-tp0367*** **)**	160	0	2	-	2	-	-	-	-
***rpiA***	217	0	1	1	0	0.00044	0.00000	0.00059	0
***tp0617***	84	0	3	0	3	0.01451	0.05193	0.00000	-
***tprI***	162	0	3	3	3	0.00947	0.00000	0.12660	0
***tp0668***	243	1	0	0	1	0.00000	0.00000	0.00000	0
***cfpA***	511	0	2	1	2	0.00152	0.00464	0.00065	7.13846
***tpf-1***	110	0	2	1	1	0.00542	0.00323	0.00612	0.52778

1 In parsimony analysis, gaps were considered a fifth character state.

2 In the *tp92* gene, residues 1531–1593, 1672–1794, 1837–2106, 2140–2334, and 2386–2502 were included in this analysis. Regions that may have undergone recombination were omitted.

**Table 4:** Summary of divergence between *T. pallidum* and *T. paraluiscuniculi*.

Type		Number
Intergenic	INDELS	5
	SNPs	13
Genic	INDELS	2
	SNPs	31

**Figure 2:**
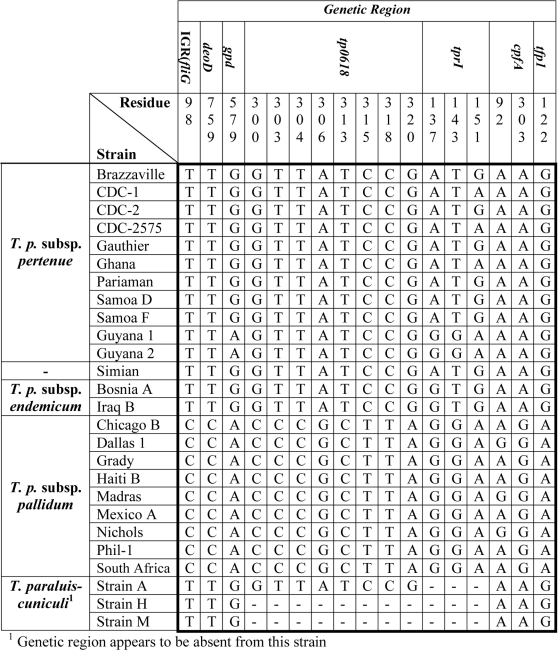
Comparison of 17 single nucleotide polymorphisms present in pathogenic *Treponema* strains, including 2 strains of *T. pallidum* subsp. *pertenue* gathered from Guyana.

A few polymorphic regions analyzed here are of special interest in light of past studies and the paucity of genetic variation described to date. Polymorphism between subsp. *pertenue* strains occurring in the first 200 basepairs of the *tprI* gene, demonstrated here, had not been described in previous studies of this gene [23],[47]. In the small region of *tprI* sequenced here, all 3 substitutions documented in *T. pallidum* strains were non-synonymous and in close proximity (Table 3). Two resulted in radical amino acid substitutions (Table S2). Variation in the *cfpA* gene had previously been reported in a study comparing the sequence of this gene in the Nichols and Haiti B subsp. *pallidum* strains [36]. We observed only one of the substitutions reported in this article, a polymorphism occurring among subsp. *pallidum* strains at position 92 (Fig. 2). However, we discovered one fixed difference between the non-venereal subspecies and subsp. *pallidum*, at residue 303 (Fig. 2). Much of the polymorphism in *tp92* was not analyzed in this study, because it fell within hyper-variable regions that were difficult to align and in which recombination could not be ruled out. Even so, in the regions included, 7 non-synonymous and no synonymous substitutions occurred among *T. pallidum* strains (Table 3). Four of these substitutions resulted in radical amino acid changes (Table S2). Finally, in IGR(*fliG-tp0027*) the presence of a SNP followed by a long homonucleotide repeat region (Fig. S1) was documented in subsp. *pallidum*, but not in the other subspecies. The net polymorphism in the former strains was predicted, on the basis of conformational stability, to form a stem-loop structure in the intergenic region between the oppositely transcribed genes *fliG* and *tp0027*. This structure, located between the predicted promoter and transcriptional start site of the operon containing genes *tp0027* and *tp0028* (Fig. S1), could attenuate transcript levels of these genes. Both of these genes are homologous to *tlyC*, which is believed to either code for a hemolysin or for a protein that regulates hemolysin production [48].

**Figure 3:**
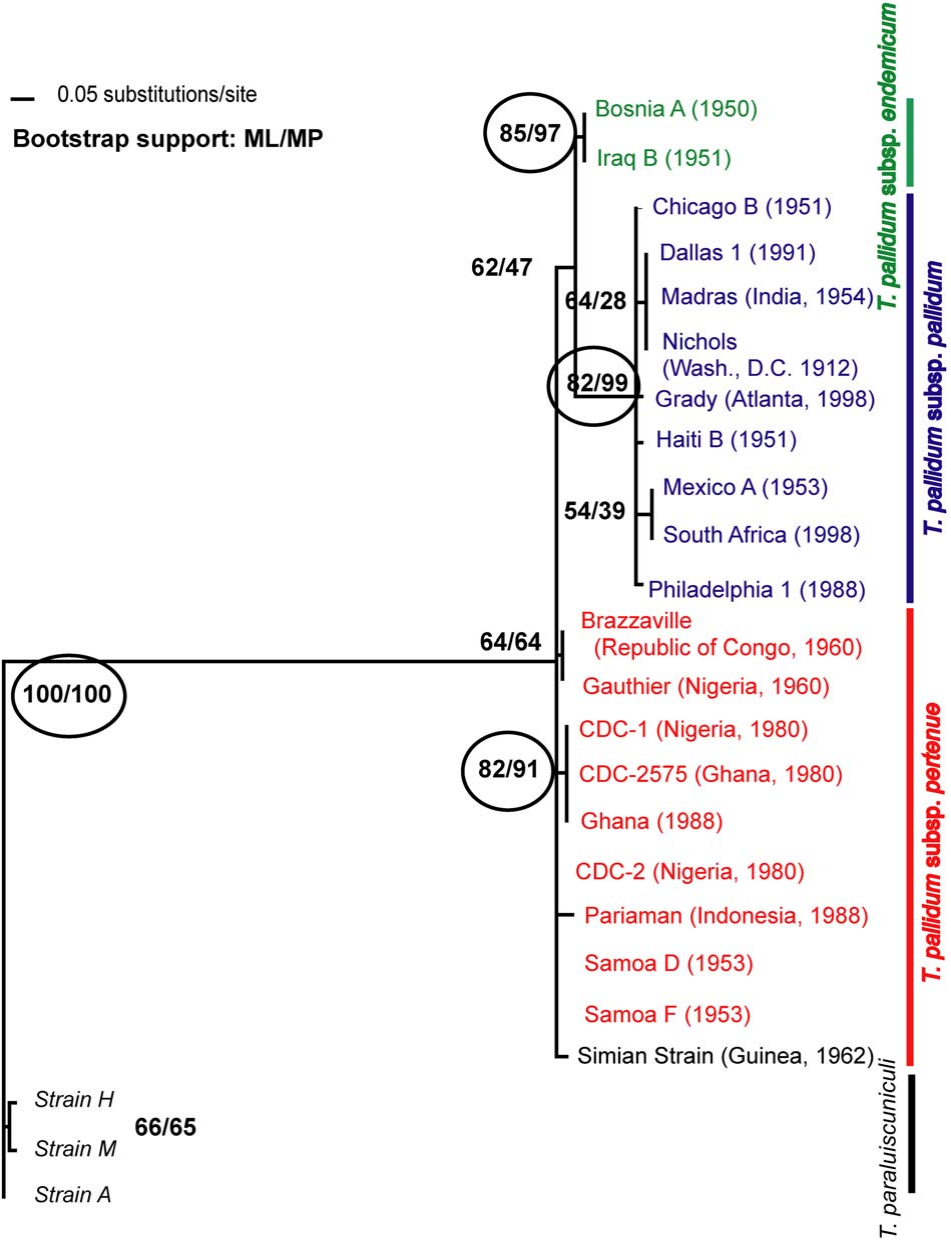
A phylogenetic tree depicting the relationships between the *T. pallidum* subspecies. This maximum likelihood tree is based on 20 polymorphic regions in the *T. pallidum* genome. Bootstrap support was estimated with 1,000 replicates in order to assess confidence at branching points and are shown within circles where values are high (>90%). Bootstrap support values for both maximum likelihood and maximum parsimony trees are shown, in that order.

A phylogenetic tree (Fig. 3), constructed using maximum likelihood and parsimony methods, demonstrated that all *T. pallidum* strains fell within a single clade. Within this larger clade, several *T. pallidum* clades with bootstrap support greater than 90% were identified. Subsp. *pallidum* and subsp. *endemicum* strains formed groupings distinct from subsp. *pertenue* strains. In addition, subsp. *pertenue* strains CDC-1, CDC-2575, and Ghana formed a clade distinct from the other strains of this subspecies, including those gathered nearby. Several clades with lower bootstrap support were also identified within the *pallidum* and *pertenue* subspecies. The subsp. *pertenue* strains occupy a basal location on the tree, indicating an ancestral position for them in the *T. pallidum* family. This basal position is supported by the average number of nucleotide differences from the *T. paraluiscunili* clade, which was lower for the subsp. *pertenue* strains (50.13) then for the subsp. *endemicum* and *pallidum* clade (55.15), or for the individual subsp. *endemicum* (51.33) and subsp. *pallidum* (56.00) clades. The genetic distance between subsp. *pertenue* and *endemicum* strains was small compared to the distance between these non-venereal subspecies and subsp. *pallidum*. The terminal position of the subsp. *pallidum* clade on the tree indicates that it diverged most recently.

The results obtained from this phylogenetic analysis were robust. Trees in which trimmed indels were weighted one-fifth the value of substitutions were found to be qualitatively indistinguishable from unweighted trees. And because many of the substitutions analyzed here came from the portion of *tp92* sequenced, a tree built without this gene was constructed (tree not shown). The major groupings described above were observed. However, without *tp92,* groupings that appeared in Fig. 3 with low bootstrap support, such as the clade containing the Mexico A and subsp. *pallidum* strain from South Africa, were not identified. Trees built using only synonymous or non-synonymous substitutions demonstrated that while the subspecies clades could be obtained by analyzing only synonymous substitutions, non-synonymous substitutions were responsible for increased phylogenetic resolution (trees not shown). That is, identification of all within-subspecies clades was dependent on non-synonymous substitutions.

**Figure 4:**
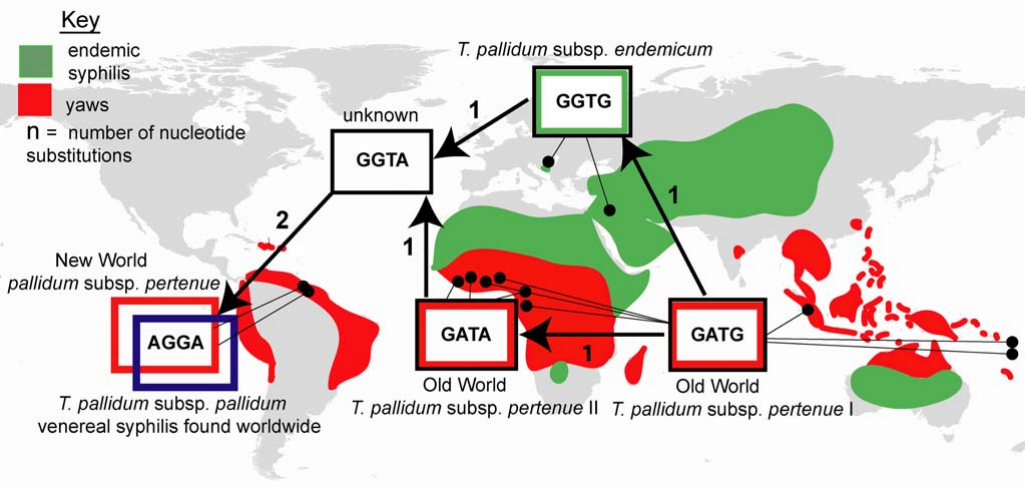
A network path for four informative substitutions shows that New World subsp. *pertenue*, or yaws-causing strains, are the closest relatives of modern subsp. *pallidum* strains. The geographical distribution of the endemic treponemal diseases circa 1900 is shown, based on a map created by Hackett [62]. Each polymorphism pattern is linked to the sites where the strains that contain it were gathered. Arrows convey the directionality of change, determined from the previously built phylogenetic tree as described in the text. The four substitutions were located in two genes located on separate sides of the genome, *tprI* and *gpd*. The locations from which subsp. *pallidum* strains were gathered are listed in Table 1.

Genetic analysis of the two subsp. *pertenue* strains collected from indigenous groups in Guyana revealed that they were the closest relatives of modern subsp. *pallidum* strains identified in this study. Only a subset of seven genetic regions could be analyzed in these strains: *IGR(fliG-tp0027)*, *deoD*, *gpd*, *tp0618*, *tprI*, *cfpA*, and *tpF-1.* These regions contained 17 SNPs at which Old World non-venereal strains differed from subsp. *pallidum* strains (Fig. 2). At 4 of the 17 SNPs examined, the New World subsp. *pertenue* strains were found to be identical to subsp. *pallidum* strains. These 4 SNPs occurred in 2 loci on separate sides of the genome, *tprI* and *gpd*. At the remaining 13 sites, the New World *pertenue* strains were identical to Old World non-venereal strains. A network path was constructed, in which these SNPs were considered in their geographic context (Fig. 4). In addition, polarity of substitutions was determined using data from the previously constructed phylogeny (Fig. 3). The tree indicated that the ancestral state of the *gpd* gene could be found in Old World subsp. *pertenue* and *endemicum* strains. In addition, polymorphism in the *tprI* gene could be used to divide *T. pallidum* into four groups: subsp. *pallidum*, subsp. *endemicum*, and two smaller groups of Old World subsp. *pertenue*. The phylogeny indicated that of the two Old World subsp. *pertenue* groups, the more recently diverged was the CDC-1/CDC-2575/Ghana one, while the other subsp. *pertenue* group was ancestral. Thus, the 4 SNP sequence present in the majority of Old World subsp. *pertenue* strains appears to have arisen first. The network path suggests that a series of substitutions led from this first group of Old-World *pertenue* strains to a second group of African subsp. *pertenue* strains and to subsp. *endemicum* strains (Fig. 4). The pattern of substitutions suggest that a hypothetical intermediate strain, arising from either the group II subsp. *pertenue* strains or *endemicum* strains, once existed and was a progenitor to both New World subsp. *pertenue* and to all subsp. *pallidum* strains. This data also suggests that the New World subsp. *pertenue* strains belong to a group distinct from the Old World subsp. *pertenue* strains, occupying a phylogenetic position somewhere between Old World non-venereal strains and modern subsp. *pallidum* strains.

## Discussion

In the past, a number of different hypotheses regarding the origin of *T. pallidum* subsp. *pallidum*, the causative agent of syphilis, have been put forth. Using new data collected in this study, we assess a number of these hypotheses.

The phylogenetic tree created in this study sheds light on the relative order in which the *T. pallidum* subspecies emerged. Subsp. *pertenue* strains gathered from central Africa and the South Pacific occupy basal positions on the tree, indicating that they most closely resemble the ancestral pathogen in humans (Fig. 3). The early emergence of these strains in human history is also supported by their low average nucleotide differences from *T. paraluiscuniculi* and their similarity to the simian strain of *T. pallidum*, which infects wild baboons. Although the simian strain could not be distinguished from subsp. *pertenue* strains using the polymorphic data in this study (Fig. 3), another study focusing on the *tpr* genes found that it was distinct from human *T. pallidum* strains examined [47]. This evidence is consistent with the hypothesis that yaws is an heirloom disease in humans, one caused by a pathogen that infected our anthropoid ancestors and has evolved with our species [49]. The presence of yaws in wild populations of our closest relatives, gorillas and chimpanzees [50],[51], further supports this theory. However, a more recent cross-species transfer event between humans and non-human primates cannot be ruled out using the available genetic data. It has been shown that inoculation with the simian strain can cause a yaws-like infection in humans [52], and it is known that infection rates are high in both humans and wild baboons in yaws-endemic areas of West Africa [53],[54]. Thus, it is possible that non-human primates serve as a source of human disease, or vice versa. Therefore, in the future it would be desirable to collect *T. pallidum* strains from various wild, non-human primate species and to sequence them at additional loci. Such information is likely to provide important information concerning the antiquity of yaws in humans.

Subsp. *endemicum* strains, gathered from the Middle East and the Balkans, diverged from subsp. *pertenue* strains at some later date, and subsp. *pallidum* strains diverged most recently, indicating that they emerged relatively recently in human history (Fig. 3). The topology of the tree is consistent with the long-held belief that treponemal disease is very old and has traveled with humans during their migrations, evolving from ancestral subsp. *pertenue*, in hot, humid regions, into subsp. *endemicum* as people settled in cooler and dryer areas, and finally into subsp. *pallidum* [9],[55]. Examination of additional variable sites in non-venereal strains from Africa and Asia may aid in pinpointing the trajectory of this pathogen family in the Old World.

The study of two yaws-causing strains from the Americas provides additional clues to understanding the history of syphilis. The genetic analysis of the two subsp. *pertenue* strains gathered in Guyana demonstrates that they are the closest relatives of venereal syphilis-causing strains identified in this study (Fig. 4). These strains are genetically distinct from Old World subsp. *pertenue* strains, having diverged more recently than Old-World non-venereal strains. The geographic analysis of strains, paired with the phylogeny, suggests a three-stage model for *T. pallidum's* dissemination and evolution. First, *T. pallidum* arose in the Old World, in the form of non-venereal infection, before spreading with humans to the Middle East/Eastern Europe, in the form of endemic syphilis, and then to the Americas, in the form of New World yaws. Second, a *T. pallidum* strain from the Americas was introduced back into the Old World, probably as a result of the European exploration of the Americas, becoming the progenitor of modern syphilis-causing strains. Third, modern subsp. *pallidum* strains disseminated from Europe to the rest of the world.

Descriptions of the clinical presentation of yaws in indigenous Guyanese patients [31] support the “transitional” position of South American subsp. *pertenue* strains, between Old World non-venereal and subsp. *pallidum* strains, indicated by the genetic comparison in this study. These yaws patients, who are inhabitants of Guyana's interior, typically present with a chancre (a chronic, painless ulcer with raised margins) similar to the type seen in venereal syphilis, though found in children and in extra-genital locations. This clinical presentation is quite different from the textbook “frambesiform” lesion characteristic of yaws in Africa and Asia. It is possible that this distinctive lesion results from differences in the pathogen genome, although host differences, both cultural and genetic, may also play a role. Though modern travel increases the probability that a strain present in one place may have been recently introduced from another, the isolation of the aboriginal population from which these strains were collected, as well as their unique genetic and clinical characteristics, makes such an event unlikely. It is possible that these strains possess some of the characteristics of subsp. *pallidum* strains while still retaining many of the features of subsp. *pertenue* strains, including a non-sexual transmission mode. The condition of the two samples precluded a complete comparative study involving these strains, but the distinct genetic make-up indicated by the available data makes further study of South American subsp. *pertenue* strains desirable, if additional samples can be gathered in the future.

Although the data suggest that venereal syphilis-causing strains arose recently, from a New World progenitor, the transmission mode of the ancestral bacterium remains unclear. The closest relatives of syphilis-causing strains in this study were non-venereally transmitted. However, because of the disappearance of endemic treponemal disease from South America it was only possible to study two indigenous strains from this continent, gathered in close proximity. It is possible that a strain even more closely related to subsp. *pallidum* once existed in pre-Columbian South America and was transmitted venereally. Paleopathologists have assessed the age distribution of treponemal infection in pre-Columbian Native American populations, in hopes of determining the mode of transmission in these civilizations. However, because permanent bone remodeling due to treponemal infection usually occurs in tertiary-stage disease, which can take many years to develop, it is difficult to determine at which age individuals typically contracted the infection in past populations, even when finds are abundant [56]. Several possible cases of congenital syphilis, believed by many to occur only in venereal infection, have been reported from pre-Columbian America, but the diagnoses remain tentative [56]. Some researchers have attempted to determine the nature of treponemal infection in past populations through statistical comparison of specific pathologies in the skeletal record. Based on this method, they assert that the treponemal infection present in the Dominican Republic at the time of Columbus's landing was more akin to venereal syphilis than yaws or endemic syphilis [57]. However, this method remains controversial, because of the limited samples from which specific pathology rates have been determined for each disease [56]. Therefore, it is not clear whether venereal syphilis existed in the New World prior to Columbus's arrival. While it is possible that Columbus and his crew imported venereal syphilis from the New World to Europe, it is also possible that the explorers imported a non-venereal progenitor that rapidly evolved into the pathogen we know today only after it was introduced into the Old World. Indeed, analysis of the changing descriptions of venereal syphilis following its appearance in Europe have led many to believe that the pathogen did evolve rapidly after its initial introduction [16]. Given the limitations of the available data, the question of whether the progenitor of modern syphilis-causing strains was venereal or non-venereal may remain unresolved.

The results of this molecular study clarify some findings of skeletal biology while obfuscating others. The virtual absence of syphilitic lesions from Pre-Columbian Old World skeletons can be explained simply in the context of this data; syphilis did not exist in these areas until the Renaissance. On the other hand, the absence of lesions typical of yaws or endemic syphilis in these areas is puzzling in light of the genetic data. If the non-venereal treponematoses arose in the Old World long before syphilis, then why isn't there more evidence of their presence? If yaws was the first form of treponemal disease, as indicated by our study, and was limited to hot, humid areas, we would expect preservation of ancient, affected remains to be poor. This may contribute to the paucity of skeletal finds.

In light of the hypotheses regarding the rapid evolution of subsp. *pallidum* in Renaissance Europe and the lack of knowledge concerning the genetic basis for the different clinical manifestations of the treponematoses, any evidence of positive selection or functional change in the *T. pallidum* genome would be of great interest. A comparison of trees constructed with only synonymous or non-synonymous substitutions emphasized the role of non-synonymous substitutions in differentiating *T. pallidum* strains. Ten of the 14 non-synonymous substitutions observed between *T. pallidum* strains occurred in just 2 genes: *tprI* and *tp92*. Similarly, 6 of 9 radical amino acid substitutions observed between *T. pallidum* strains occurred in these 2 genes (Table S2). Given the evidence for the role of the TprI and Tp92 proteins in pathogenicity [22],[58],[59], the substitutions clustered in the regions of these genes that were sequenced may hint at positive selection. Sequencing the entirety of these genes in many strains of *T. pallidum* may be worthwhile, in order to better assess this possibility. Similarly, since *tp0027* and *tp0028* are both homologous to *tlyC*, a gene that either encodes a hemolysin or regulates a cryptic one in *E. coli* [48], a difference in transcript level between subspecies could affect pathogenesis. To this end, a regulatory function for the predicted stem-loop structure in IGR(*fliG-tp0027*) in subsp. *pallidum* could be tested. Transcript levels of *tp0027* could be characterized in the different subspecies; in addition, the two IGR variants discovered in this study could be placed upstream of a reporter gene in a genetically tractable bacterium, such as *E. coli*, in order to directly examine the effect of the stem-loop structure on transcript levels.

Our conclusions regarding the history of *T. pallidum* differ from those drawn in a recent comparative study of the *tpr* gene family [23], in which it was asserted that the times of emergence for the pathogens that cause endemic syphilis, yaws, and syphilis were similar and dated to sometime later than the emergence of modern humans but earlier than the Renaissance. We propose several reasons for why the conclusions arrived at in the two studies diverge. The strains collected from Guyana in this study played an integral role in our analysis, because they were most closely related to syphilis-causing strains and helped establish the geographic trajectory of *T. pallidum* evolution. In addition, in the genetic regions we examined, recombination was much less common than in the majority of the *tpr* genes, if it was present at all. Our data suggest that the *T. pallidum* genome is evolving in a largely clonal manner amenable to phylogenetic analysis, with the exception of frequent intra-gene conversion events confined within the members of the *tpr* gene family [23]. Finally, because *T. paraluiscuniculi* was extremely similar to *T. pallidum* at all but one of the sequences examined (*tprI* is missing in the former species), it was easy to create alignments and to assess the directionality of mutations in this study. The evolutionary pathway between the *tpr* genes of *T. paraluiscuniculi* and *T. pallidum* is more complicated and much harder to interpret.

This study has some obvious limitations. The level of polymorphism found between *T. pallidum* strains is quite low, as suggested by previous studies. For this reason, the level of resolution in the phylogenetic tree is relatively poor. It is likely that polymorphism data from the rest of the genome will clarify the topology of this tree, including the relationship between subsp. *endemicum* and subsp. *pallidum* strains, which are grouped together in this study with low bootstrap support, and the position of subsp. *pertenue* strains, which occupy a basal position on the tree but group together only by default. Similarly, the close relationship between subsp. *pallidum* strains and New World subsp. *pertenue* strains described in this paper hinges on the analysis of only 4 SNPs. Three of these SNPs were found in a single gene, *tprI*. The close proximity of these non-synonymous SNPs within the gene, the non-linear relationships of the strains indicated by the substitutions (Fig. 4), and the evidence that the TprI protein may be involved in pathogenesis, suggest that *tprI* may not be evolving neutrally. For this reason, it is unlikely that these SNPs have accumulated in a clockwork manner. Instead, the information that can be gleaned from this gene is limited to the relative order in which evolutionary events occurred.

The large-scale comparative genetic studies possible on the pathogens that cause diseases such as malaria and anthrax will never be possible in *T. pallidum*, because of the disappearance of the non-venereal treponematoses and the strains that cause them [60],[61]. The prevalence of yaws in Guyana, the last country in South America in which yaws has been documented in recent years, has decreased annually, and surveys carried out by our group in 2006 and 2007 in endemic yaws territory demonstrated no active cases of the disease. Analysis of South American strains is necessary in order to assess the relationship between subsp. *pallidum* and non-venereal treponemal strains. Because it is not clear whether an opportunity to examine such strains will arise again, the results presented in this paper are of special importance in the debate over the origins of treponemal disease.

In conclusion, in this study we found that syphilis-causing strains evolved relatively recently in human history and that the closest relatives of subsp. *pallidum* were yaws-causing strains from the New World. When this genetic data is combined with extensive documentary evidence that syphilis appeared in Europe for the first time around 1495 [12] and the apparent absence of skeletal signs of syphilis in pre-Columbian Europe and North Africa, the Columbian hypothesis for syphilis's origin gains new strength.

## Supporting Information

Figure S1 Predicted stem-loop structure observed in all *Treponema pallidum* subsp. *pallidum* strains in IGR(fliG-tp0027). All nucleotides preceding the transcription start site are shown, with the predicted polymerase binding site in red and the putative hemolysin genes tp0027 and tp0028 following the intergenic region. The homonucleotide repeat that allows this stem-loop formation is absent in subsp. *endemicum* and *pertenue* strains, and its presence may affect transcription of the genes ahead.
(0.04 MB DOC)

Table S1 Primers and annealing temperatures used in this study.
(0.07 MB DOC)

Table S2 Polymorphism table.
(0.07 MB XLS)

Table S3 Polymorphism table including excluded regions.
(0.09 MB XLS)
