# Supplementary material for: On the Origin of the Treponematoses: A Phylogenetic Approach
Source: PLoS Negl Trop Dis. 2008 Jan 15;2(1):e148. doi: 10.1371/journal.pntd.0000148 (PMC2217670; doi:10.1371/journal.pntd.0000148)
Supplement: Table S1 — Primers and annealing temperatures used in this study. (0.07 MB DOC) [file pntd.0000148.s002.doc]

| **Genetic Region^1^**  Genbank ID | **Primers^2^** | **Annealing Temperature** |
| --- | --- | --- |
| **IGR(*fliG-tp0027*)** | **F**:GCCACGAGGAGATTCGGTTCTATTC  **R**:CCGCAGCAGAGAACAACATGGA | 55° |
| ***cysS (tp0091)***  3322350 | **F**:GTGCAGGAGGGAACGTGTAT  **R**:TACGGGTAAACCACAGCACA | 55° |
| ***tp0106***  3322369 | **F**:ATCTCGTGCGCACTCCTATT  **R**:GAGAAACCATGAGCGAAAGG | 55° |
| ***tp0123***  3322389 | **F**:GCTATGTGCGCGTATGAGAA  **R**:ATTGGGGAAAAGACGAATCA | 55° |
| **IGR(*tp0129-tp0130*)**  ***tp0130***  3322397 | **F**:CCATACCGGGAAGTTGATGT  **R**:TCCTGCTGCTCCTACGAAGT | 55° |
| **IGR(*tp0135-tp0136*)** | **F**:CGAATGCAGTACATCGTGTTG  **R**:CAGCCCAGTCGTCCTTTATC | 55° |
| ***deoD (tp0170/ pfs)***  3322437 | **F**:GGTTACCAGAAAGGGCGTATTCC  **R**:CGACCATTACACGACCATC | 55° |
| ***gpd* (*tp0257/ glpQ*)**  2611763 | **F**:AAGAACTTTCCCTCCTCCGTGC  **R**:CGTTTGATACGCTTCAGCTCG | 60° |
| ***tp92* (*tp0326*)**  3322602 | **F**:AGAGCCTGAAGCTCGGGTAT  **R**:ACCGTGAACGACAACACAAA | 55° |
| ***tp0347***  3322629 | **F**:ATCTGCTGAAAGGCTCACAGTACAGC  **R**:GGAACGTGCATGTGCTGCCC | 60° |
| **IGR(*cheY-tp0367*)** | **F**:TAAAGTGAGGGCGGATGTGT  **R**:GCTGAGGGAGACACCACTTC | 55° |
| ***rpiA (tp0616)***  3322914  ***IGR(rpiA-tp0617)***  ***tp0617***  3322915 | **F:**CCGAAGCGGTAAGAAAAAAAG  **R**:GCTACCGTTTCGTTTGGC | 55° |
| ***tp0618***  3322924 | **F**:CGAACACACCGAACCCTCATTG  **R**:AGTATTTACCTCCCCGTTATGCG | 60° |
| ***tprI* (*tp0620*)**  3322917 | **F**:TGTGCTTTGACACCAACGGC  **R**:TGGGAACAGGGTATGCTCTCTG | 60° |
| ***tp0668***  3322970 | **F**:GTCGGTTCGGTTTCTGTGTT  **R**:GGACGCACGGTAACTCAAAT | 55° |
| ***tp0740***  3323047  **IGR(*tp0740-tp0741*)**  ***tp0741***  3323048 | **F**:ATACCTAGGTGGCGAAAGCA  **R**:GTACCTTCCCCAGTTCGTGA | 55° |
| ***cfpA* (*tp0748*)**  2611117 | **F**:GAGTCCCAATGTGTTTCATCC  **R**:GAACGCACACTTGACTACCG | 55° |
| ***tmpB (tp0769)***  2611387 | **F**:CGTAATTTCTCGCTCGTATCC  **R**:CGGCAGAGGACGGTATTTCAC | 58° |
| **IGR(*tex-fld*)**  ***fld (tp0925)***  3323245 | **F**:CCGTTTGCCCAGTTCTTCAAAG  **R**:TCAAAAGTACCAACGTCGAAGTCC | 58° |
| ***tp0989***  3323313  **IGR(*tp0989-tp0990*)**  ***tp0990***  3323314 | **F**:GCATGCTAAAGGGAAACCTG  **R**:TCTGGCTCATTCCCAAGAGT | 55° |
| ***tpf-1* (*tp1038*)**  2610937 | **F**:GAAAAAAATACCACAGCACCGC  **R**:CAATGCCGTAGATGTGCCAGTG | 60° |

**^1^** Genes are identified by both commonly used names, when present, and the gene number in the *T.* *pallidum* genome. Intergenic regions (IGRs) are identified by the genes between which they fall. Where amplicons contained multiple genetic regions, they are listed on separate lines.

^2^ Used for both PCR and sequencing.
