# Supplementary material for: On the Origin of the Treponematoses: A Phylogenetic Approach
Source: PLoS Negl Trop Dis. 2008 Jan 15;2(1):e148. doi: 10.1371/journal.pntd.0000148 (PMC2217670; doi:10.1371/journal.pntd.0000148)
Supplement: Supplementary file 2 [file pntd.0000148.s001.doc]

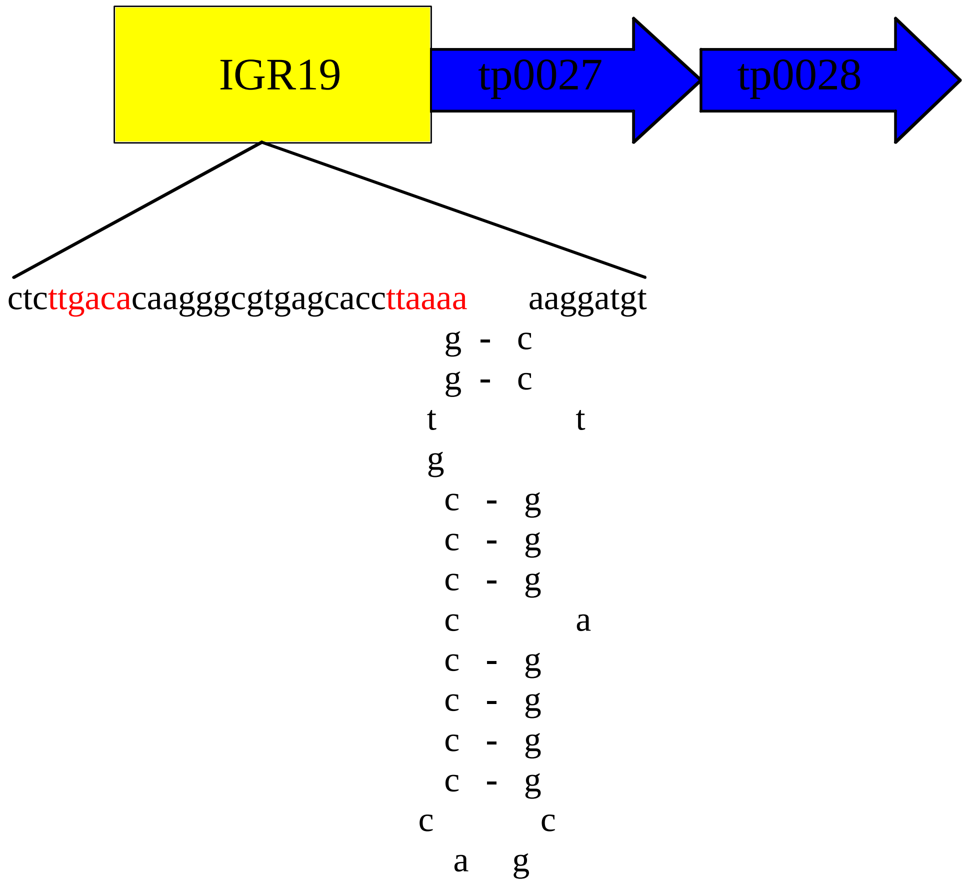
IGR19
tp0027
tp0028
ctcttgacacaagggcgtgagcaccttaaaa aaggatgt
g - c
g - c
t t
g
c - g
c - g
c - g
c a
c - g
c - g
c - g
c - g
c c
a g
